# The Immune Microenvironment of Malignant Pleural Mesothelioma: A Literature Review

**DOI:** 10.3390/cancers13133205

**Published:** 2021-06-26

**Authors:** Anne-Laure Désage, Georgia Karpathiou, Michel Peoc’h, Marios E. Froudarakis

**Affiliations:** 1 Department of Pulmonology and Thoracic Oncology, North Hospital, University Hospital of Saint-Etienne, 42055 Saint-Etienne, France; Anne-Laure.Desage2@lyon.unicancer.fr; 2 Pathology, North Hospital, University Hospital of Saint-Etienne, 42055 Saint-Etienne, France; georgia.karpathiou@chu-st-etienne.fr (G.K.); michel.peoch@chu-st-etienne.fr (M.P.)

**Keywords:** malignant pleural mesothelioma, immune microenvironment, tumour-associated macrophages, tumour infiltrating lymphocytes, exhausted T-cells, immune checkpoints

## Abstract

**Simple Summary:**

Malignant pleural mesothelioma is a rare and aggressive tumour, associated with asbestos exposure. Current therapeutic approaches for malignant mesothelioma are mainly based on systemic chemotherapy with a median overall survival of less than two years. In the setting of immunotherapy development, there is a need to better understand the immune microenvironment of malignant pleural mesothelioma. In this review, we aim to synthetize the recent advances in knowledge on the immune microenvironment of malignant pleural mesothelioma. This literature review shows that the immune microenvironment of malignant pleural mesothelioma is highly heterogeneous and can be considered as mainly immunotolerant or immunosuppressive. Better understanding of this immune microenvironment is particularly relevant to target molecular vulnerabilities and develop new treatment strategies.

**Abstract:**

Malignant pleural mesothelioma (MPM) is a rare and aggressive tumour with a poor prognosis, associated with asbestos exposure. Nowadays, treatment is based on chemotherapy with a median overall survival of less than two years. This review highlights the main characteristics of the immune microenvironment in MPM with special emphasis on recent biological advances. The MPM microenvironment is highly infiltrated by tumour-associated macrophages, mainly M2-macrophages. In line with infiltration by M2-macrophages, which contribute to immune suppression, other effectors of innate immune response are deficient in MPM, such as dendritic cells or natural killer cells. On the other hand, tumour infiltrating lymphocytes (TILs) are also found in MPM, but CD4+ and CD8+ TILs might have decreased cytotoxic effects through T-regulators and high expression of immune checkpoints. Taken together, the immune microenvironment is particularly heterogeneous and can be considered as mainly immunotolerant or immunosuppressive. Therefore, identifying molecular vulnerabilities is particularly relevant to the improvement of patient outcomes and the assessment of promising treatment approaches.

## 1. Introduction

Malignant pleural mesothelioma (MPM) is a rare and aggressive tumour that develops from mesothelial cells. In most cases, it is related to asbestos exposure. Indeed, the relationship between asbestos exposure and MPM has been proven since 1960 and is characterized by a long latency period of 30 to 40 years [1,2,3]. The incidence of MPM seems to have reached a plateau in the USA, whereas, in most European countries, the estimated peak of incidence is expected in the near future [4,5,6].

**Figure 1:**
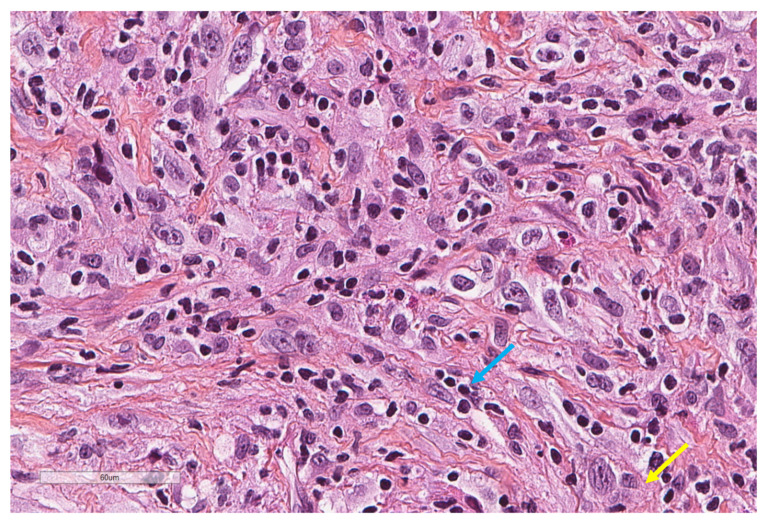
An epithelioid malignant mesothelioma showing large tumour cells (yellow arrow) intermixed with smaller lymphocytes (blue arrow) (X400).

Histologically, three subtypes of MPM can be individualized: epithelioid mesothelioma (Figure 1), which is the most frequent (60% of cases), sarcomatoid mesothelioma (10% of cases), and biphasic mesothelioma, which is composed of both epithelioid and sarcomatoid histologies (20% of cases), with 10% of cases remaining indeterminate [7,8]. Histologic subtype is considered as a prognostic factor, and it is, therefore, an important consideration. Indeed, Curran D. et al. demonstrated that sarcomatoid sub-type, as well as poor performance status, high white blood count and male gender, are associated with poor prognosis [9]. Moreover, in the epithelioid subtype, mitotic count, necrosis, nuclear atypia and nucleoli size were found to be independent prognostic factors of overall survival [10].

Asbestos carcinogenesis is mainly due to four main processes [7,11]. First, asbestos fibres penetrate deep in the lung depending on their length and width. It is known that the thicker and longer the fibres are, the more deeply they would advance into the lung [11]. As a result, they can reach the pleura after inhalation and cause repeated cycles of pleural irritation, inflammation, and repair [12]. Indeed, an interaction exists between asbestos fibres with mesothelial and inflammatory cells, releasing macrophages, cytokines and growth factors, which promote chronic inflammation and tumour growth [13,14]. Then, asbestos fibres may disrupt mitosis and induce chromosomal damage and strand breaks [15]. Asbestos fibres may also induce the production of iron-related reactive oxygen species, which results in DNA damage [16]. Finally, asbestos fibres induce phosphorylation of the Mitogen-Activate Protein Kinase (MAPK) and Extracellular signal Regulated Kinase (ERK) pathways in mesothelial cells, which are implicated in cell proliferation [17]. Recent evidence on the carcinogenesis of asbestos showed that asbestos exposure seems to be associated with a decreased antitumour immunity. Indeed, it has been showed that CXCR3 (i.e., the chemokine receptor on the surface of T helper cells) and the production of Interferon gamma (IFN-γ) were reduced in peripheral CD4+-cells of asbestos-exposed patients [18]. Moreover, in vitro experiments on MT-2 human polyclonal T-cells with chronic exposure to asbestos fibres revealed that regulatory T-cells have enhanced functions through high expression of interleukin-10 and Transforming Growth Factor-betta (TGF-β) [19].

Although the relationship between asbestos exposure and MPM has been proven for a long time, a total of only 17 incident cases of malignant mesothelioma were diagnosed among 5287 patients previously exposed to asbestos during a 7-year follow-up study [20]. This highlights the individual susceptibility to asbestos and the fact that MPM is a complex tumour implying molecular, genetic and epigenetic alterations [14]. Molecular abnormalities involved in the development of MPM are germline BAP1 (breast cancer 1-BRCA1-associated protein (1) mutation, resulting in the loss of expression of BAP1, described in familial cases of mesothelioma [21]. In addition, somatic BAP1 alteration occurs in almost 60% of sporadic MPM [22]. Genetic patterns may favour the development of MPM as assessed by the homozygous deletion of CDKN2A or loss of NF2 (Neurofibromatosis type (2) [12,23,24,25]. Furthermore, alterations in the Hippo pathway, mTOR and chromatin modifiers have been identified [26].

Nowadays, the first-line chemotherapy for MPM is the combination of cisplatin with pemetrexed, since this combination showed efficacy versus cisplatin alone [27,28]. Indeed, only a few patients are candidates for surgery. In 2016, the MAPS-1 (Mesothelioma Avastin Cisplatin Pemetrexed Study) randomized trial showed a significant benefit in overall survival (OS) (18.8 vs. 10.1 months, *p* = 0.01) and progression-free survival (PFS) (9.2 vs. 7.3 months, *p* = 0.001), when adding bevacizumab to cisplatin-pemetrexed [29] and, therefore, the ERS/ESTS/EACTS/ESTRO 2020 guidelines suggested that bevacizumab should be proposed in combination with cisplatin-pemetrexed as first-line treatment [30]. In fact, no chemotherapy regimen has been proven to be efficient beyond first-line chemotherapy. Immunotherapy is actually an approach to cancer treatment in which endogenous immune cells are harnessed to destroy tumours, and many clinical trials are currently ongoing in this field. Therefore, better understanding of the immunological microenvironment of MPM is of particular interest to target the molecular vulnerabilities associated with its development. In this review, we will detail the recent biological advances in the immunological microenvironment of MPM.

## 2. Innate Immune Cells

### 2.1. Tumour-Associated Macrophages (TAMs)

**Figure 2:**
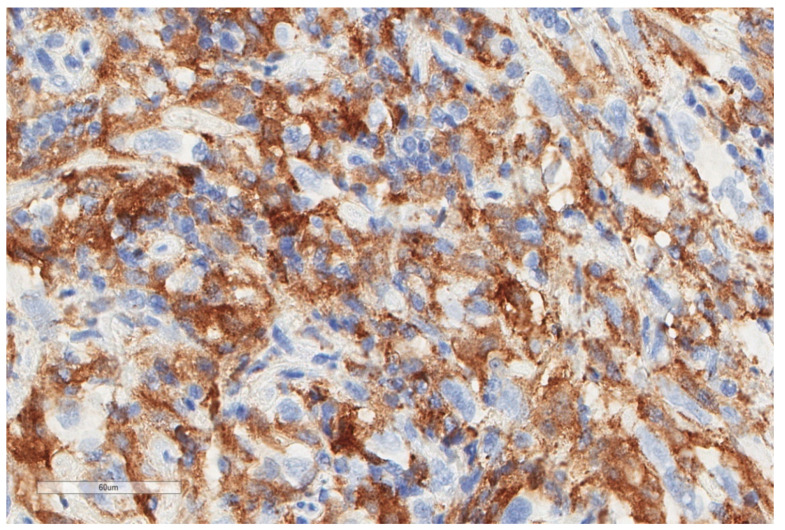
The same focus of epithelioid mesothelioma as that shown in Figure 1, stained for CD163, revealing numerous macrophages (X400).

Innate immune cells, and, more specifically, macrophages are involved at an early stage of the immune response following asbestos exposure [31,32]. Indeed, it was demonstrated in vitro that asbestos induced the secretion of Tumour Necrosis Factor-alpha (TNF-α through the activation of macrophages [32]. Human mesothelial cells express TNF-α receptor I, which leads to the activation of the NF-κB signalling pathway, and thus, favour cell proliferation and resistance to asbestos cytotoxicity [32]. This mechanism may be involved in tumorigenesis of MPM as these cells could constitute a pool of cells that survive asbestos exposure and ultimately present malignant transformation. Moreover, macrophages (CD68+) as well as natural killer (NK) cells (CD56+), have been demonstrated to constitute the main part of inflammatory cells’ infiltration in MPM [33]. According to Burt et al., macrophages account for 27% of the cellular infiltrate on tissue sections of epithelioid (Figure 2) and non-epithelioid mesotheliomas [34]. High standard deviation indicates heterogeneity in macrophage infiltration [34,35].

**Figure 3:**
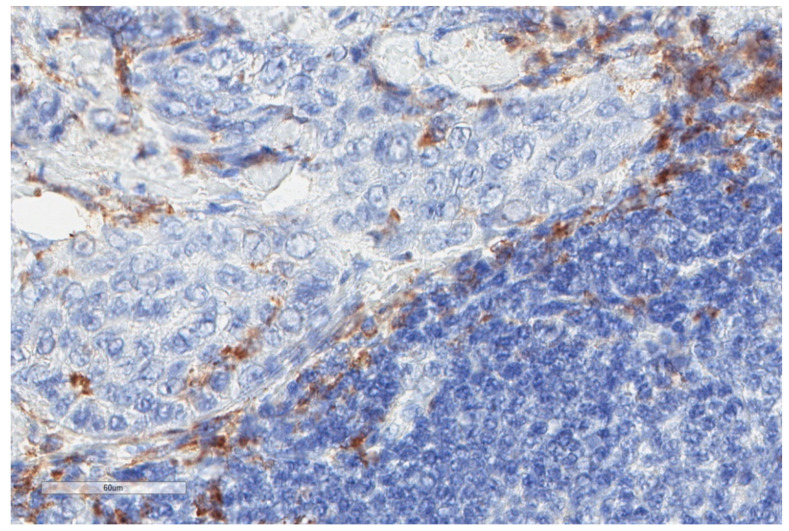
Another case of epithelioid mesothelioma stained for CD163 showing much lower numbers of positive macrophages (X400).

Macrophages are phagocytic cells that are derived from circulating monocytes. Macrophages can be present within the tumour and are called tumour-associated macrophages (TAMs) [35,36]. Interestingly, TAMs may constitute prognostic factors. Schematically, they can be divided into M1-macrophages with anti-tumour activity, and M2-macrophages (CD163+), which are characterized by a pro-tumour activity through the secretion of several cytokines (IL-1, IL-6, IL-10, Vascular Endothelial Growth Factor VEGF and TGF-β) [35,36]. M2-macrophages have been found to be highly present in both pleural effusion and tissue samples of patients with an MPM (100% of patients with M2-macrophages expression > 41% in pleural fluid and 44% in pleural tissue, respectively) [37]. M2-macrophages are important cells as they are associated with the immune-suppressive function of CD8+ cells through the secretion of arginase and several cytokines [35,36]. Ujiie et al. [38] were interested in TAMs’ and TILs’ ratio within the tumour. Patients with high CD163+ TAMs and low CD8+ TILs had worse prognosis (adjusted HR (95% CI) 1.64 (1.01–2.66); *p* = 0.044), whereas those with low CD163+ (Figure 3) TAMs and high CD20+ B cells expression had better prognosis (adjusted HR (95% CI) 1.64 (1.10–2.44); *p* = 0.015) than other groups [38].

Immunohistochemistry analysis from samples of epithelioid mesothelioma patients treated with induction chemotherapy and surgery, or chemotherapy alone, revealed that CD68 (i.e., pan-macrophage marker) and CD163 (M2-macrophage marker) were not differentially expressed in these two treatment groups [39]. Of note, the CD163/CD68 ratio was negatively correlated with OS in both groups of patients (Pearson’s r −0.72, *p* < 0.05). No significant correlation was found in the surgery group; thus, CD163/CD68 could be used as an interesting prognostic biomarker but cannot be used as a predictive biomarker after surgery [39]. Burt et al. showed that high preoperative monocyte count is negatively correlated with OS in both epithelioid and non-epithelioid mesotheliomas [34]. Higher density of TAMs was significantly associated with poorer prognosis in the non-epithelioid group in comparison with a lower density of TAMs (adjusted HR (95% CI) 1.10 (1.03–1.18); *p* = 0.005). Such correlation was not found in the epithelioid group [34]. Finally, expression of CD68+ stromal macrophages has been found to be correlated with the number of stromal T regulatory cells (R = 0.410; *p* = 0.002), thus suggesting that M2-macrophages affect the adaptative immune response [40].

### 2.2. Dendritic Cells

Other evidence suggests that the innate immune response in MPM is deficient, particularly the capacity to process antigen presentation. Dendritic cells (DCs) are important antigen-presenting cells, which are divided into myeloid dendritic cells (CD11c+, CD11b+, CD13+ and CD33+) and plasmacytoid DCs (CD123+, CD303+ and CD304+) [41]. Myeloid DCs are circulating DCs originating from CD34+ progenitors or CD14+ monocytes. Immature DCs are able to capture antigens, which enable the expression of peptide-major histocompatibility complex type II (MHC) after their maturation in the lymph node. Through the secretion of IL-12, myeloid DCs induce polarization of naïve CD4+ T-cells into Th1 cells and, thus, induce a specific immune response [41]. Cornwall et al. showed that mesothelioma patients have lower amounts of circulating myeloid DCs in comparison to healthy-age and gender-matched controls [42]. DCs also seem to be functionally deficient in mesotheliomas, as assessed by the lower expression of costimulatory (CD40, CD80 and CD86) and MHC molecules in comparison with healthy controls [42,43]. Similarly, Hegmans et al. detected rare expression of DCs in mesothelioma cells and pleural fluids from patients with a mesothelioma [33]. Such findings highlight the fact that mesothelioma patients may have a reduced capacity to process antigen presentation. In order to counteract this deficiency in antigen-process presentation, Hegmans et al. investigated pulsed-DCs using pre-clinical models [44]. Indeed, they showed that pulsed DCs, with AB1 tumour mesothelioma cell line lysate, AB1-derived exosomes or ex vivo AB1 tumour lysate, could prevent the outgrowth of mesothelioma following tumour implantation in a mouse model [31,44].

Finally, the infiltration of DCs might be influenced by treatment. CD11c+ and CD303+ DCs, as well as CD64+ macrophages, were found to be highly expressed on pleural and ascites fluids collected from MPM patients after chemotherapy (43.4% and 38.8% of viable cells, respectively) [45]. This highlights that chemotherapy could influence the immune microenvironment.

### 2.3. Natural Killer Cells (NKs)

Although NKs have been found to constitute a major part of inflammatory cells’ infiltration in MPM [33], it seems that—similar to DCs—NKs present functional alterations in MPM [46]. NKs are able to detect and kill cells that have lost or under-expressed human-leukocytes antigen (HLA) class I, which is a common feature observed in tumour cells and virus-infected cells [47]. Generally, NKs are divided into two sub-sets: (1) CD56dim NKs, which are more cytotoxic and express higher levels of Ig-like NK receptors and CD16; (2) CD56bright NKs, which produce abundant cytokines but lower cytotoxicity [48]. CD56bright NKs are CD16dim or CD16- [48]. NKs express various activating (NKG2D, 2B4, NKp46) or suppressive (NKG2A, NKG2B, killer cell immunoglobulin-like receptors, CD94) receptors on their surface, which contribute to the activation or suppression of cytotoxicity [49,50]. In the case of the binding of the activating receptor with the ligand of target cells, the Src dependent kinase pathway is phosphorylated, and, as a result, degranulation occurs with the release of perforin and granzyme. Finally, NKs can also interact with DCs as they can kill immature DCs and promote DCs’ maturation through IFN-γ and TNF-α secretion [49].

In vitro experiments using human NK cell lines YT-AI cultured with chrysotile showed that NKs have impaired functions [49]. Indeed, low expression of cell surface activating receptors NKG2D and 2B4 was observed. This impaired the cytotoxicity of NKs in vitro, which occurred five months after the beginning exposure to chrysotile. Likewise, the cytotoxicity of peripheral mononuclear cells from the peripheral blood of patients with a malignant mesothelioma was assessed against K562 cells (immortalised myelogenous leukemia cell line). The authors showed that patients with an MPM have lower cytotoxicity of NKs in comparison with healthy-matched controls, as assessed by a decreased expression of the activating receptor NKp46 [49]. As a result, these experiments showed that NKs have impaired functions, partly due to asbestos exposure. Interestingly, pre-clinical models using alpha-galactocysylceramide (α-GC)—an immunoenhancing agent—have been developed [51]. Indeed, NKs recognize α-GC through CD1d, which results in NKs’ activation. In CD1d wild-type xenograft models, after sub-cutaneous injection of AB12 cell lines—a murine malignant mesothelioma cell line derived from asbestos-induced tumour in Balb/c mice [52]—tumour growth was decreased by the combination of α-GC following each cycle of cisplatin [51]. Consistent with this observation, Ki-67 was significantly lower with this combination compared to α-GC alone. On the contrary, such efficacy was not observed in CD1d knock-out mice [51]. More interestingly, the authors observed that the number of NKs increased in tumour and peripheral blood of wild-type mice treated with cisplatin and α-GC combination compared to cisplatin or α-GC alone. In addition, cytolytic enzymes, such as granzyme B, and cytokines such as IFN-γ, were also significantly increased [51]. Such findings in pre-clinical models highlight that the immune response could be enhanced through increased activity of NKs. Ki-67 is a non-histonic nuclear protein that is expressed in all cell-cycle phases, except for the G0 phase, and the degree of Ki-67 detection by immunostaining is, therefore, indicative of the degree of cell proliferation. High proliferation index, as expressed by Ki-67, was an independent factor of poor survival in patients with epithelioid mesothelioma in a cohort of 187 patients [53].

Sottile et al. characterized immune cell profiles of circulating NK cells of 27 MPM patients (patients enrolled in MESOT-TREM 2012 and DETERMINE trials) with healthy-matched controls [54]. Most of the patients expressed a high proportion of NK-cell-specific markers [54]. At baseline, the authors observed that MPM patients presented disrupted ratios of CD56bright and CD56dim NKs, with a trend towards reduced frequencies of CD56dim NKs and increased frequencies of CD56bright NKs, compared to healthy controls. Furthermore, CD56bright NKs had reduced expression of CD62L in MPM patients, which is responsible for the migration of lymphocytes to lymph nodes [54]. Interestingly, after tremelimumab treatment (anti-CTLA-4 antibody), this ratio returned to physiological conditions, with a higher proportion of CD56dim NKs [54]. Moreover, high DNAM-1+ CD56dim NKs (DNAM-1 is an adhesion receptor of NKs) frequency and high expression of NKp46 on CD56dim NK cells was associated with an improved OS at baseline and after tremelimumab treatment [54].

### 2.4. Innate Lymphoid Cells (ILCs)

Innate lymphoid cells (ILCs) are tissue-resident cells with a lack of antigen-specific receptors [55,56]. ILCs are divided into five groups according to cytokine production: NK cells, ILC1 (IFN-γ production as NK cells), ILC2 (IL-5, IL-13), ILC3 (IL-22, IL-17) and lymphoid tissue-inducer cells [55,56].

Tumino et al. assessed the presence of ILCs in pleural effusions of 54 patients, including 33 samples from patients with an MPM [57]. ILC3 was more highly expressed (71%) than ILC1 and ILC2 (12.1% and 16.9%, respectively). Interestingly, the authors assessed that ILCs were functionally active, typically through the expression of cytokines. In addition, it was found that NKs and ILC3 expressed PD-1, whereas tumour cell lines derived from pleural effusion expressed PD-L1 [57]. Such findings highlight that the tumour microenvironment may harness NKs and immune lymphoid cells to produce an anti-tumour response.

Other innate immune cells: Similarly, to DCs, mast cells, neutrophils and other myeloid-derived suppressive cells (MDSCs), seem to be poorly expressed in the immunological microenvironment of MPM [58]. However, their immunosupressive role is actually under investigation, with studies reporting that high concentrations of MDSC seem to supress TILs and, therefore, might be related to poor OS and PFS [37].

Overall, these studies highlight that an immunosuppressive microenvironment (i.e., CD163+ M2-macrophages, dysfunction of DCs and NKs) can be observed in MPM and, thus, contributes to the escape of the tumour from immune surveillance (Table 1).

## 3. Adaptive Immune Cells

### 3.1. Tumour-Infiltrating Lymphocytes

**Figure 4:**
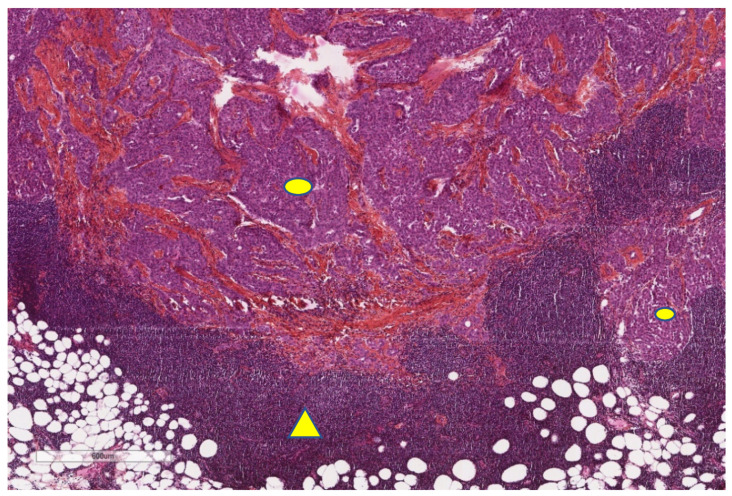
Important infiltrate of lymphocytes (triangle) surrounding tumour (ovals) in the same case of malignant mesothelioma shown in Figure 3 (X40).

Tumour-infiltrating lymphocytes (TILs) (Figure 4) assessment has been implemented for a few years, as their prognostic value has been revealed in several solid cancers [59]. As previously explained, DCs have properties of antigen-presentation to naïve T-cells. The activation of naïve T-cells leads to the generation of effector T-cells [60]. T-lymphocytes express different receptors on their membrane surface: CD28, LF-A1, TCR, CD2, CD5 and CD4 or CD8. According to CD4+ or CD8+ expression, we can differentiate effector cells with helper properties (CD4+) or cytotoxic properties (CD8+) [59]. Helper CD4+ T-cells (MHC type II), through the secretion of IFN-γ and IL-2, enhance the cytotoxic activity of CD8+ T-cells [59]. Helper CD4+ T-cells also have direct properties of cytotoxicity through the secretion of IFN-γ, TNF-α and cytotoxic granules. CD8+ T-cells (MHC type I) induce apoptosis of tumour cells or infected cells through the secretion of cytotoxic cytokines (IFN-γ and TNF) and toxic molecules such as perforin and granzyme [60]. Memory T-cells are responsible for secondary response: 1) central memory T-cells remain in lymphoid tissues and express CCR7 and CD45RO on their surface, and 2) memory T-cells in tissues lack of CCR7 expression and express CCR3, CCR5 and CD45RO on their surface [60]. Regulatory T cells (Tregs), which express the CD4, CD25 and FOXP3 phenotypes, are found in tumour microenvironments and induce immune tolerance as they downregulate CD4 helper and CD8 cytotoxic T cells. Moreover, Tregs are also known to decrease DCs’ antigen presentation [61]. As a consequence, high infiltration of Tregs within the tumour is associated with a poor prognosis [61].

Evidence suggests that in MPM, adaptative immune cells—CD4+ helper T-cells and CD8+ cytotoxic T-cells—are mainly expressed in the stromal compartment of the tumour, whereas regulatory T-cells border the tumour cells [43,58]. TILs are highly expressed in both peritumoral and stromal compartment of epithelioid and sarcomatoid MPM [62]. It is estimated that TILs represent an average of 20 to 42% of the immune cell infiltration in MPM [58].

B cells are also considered an integral component of the adaptive immune system. Their complicated functions will prominently affect anti-tumor response and be widely used for different clinical applications. B cells may act as antibody-generator or antigen-presenting cells (APCs). The antibodies secreted by B cells may bind to tumour antigens and amplify the adaptive immune response [63].

The Ab-based therapy of tumours is commonly applied in patients with hematological malignancies and solid tumours through direct or indirect mechanisms. The therapeutic functions can be emphasized by targeting certain surface Ags of lymphocytes, such as CD40 [64], CD20 [65], CD19 [66], CD73 [67], CTLA-4 or PD-1 immune checkpoints [68], blocking the binding of specific ligands, and perturbing the EGFR and HER2 signaling pathways [69,70].

In combination with conventional cancer therapies, Ab-based therapies that target these factors can stimulate anti-tumor response and improve clinical efficacy. B cells can also promote anti-tumor immunity through providing Ags to both CD4+ and CD8+ T cells [71] or through cross-presentation of Ags to other APCs [72].

B cells work also as cytokine producers, releasing interleukin (IL), interferon (IFN), and other cytokines that enhance anti-tumor immunity [63]. By producing these cytokines, B cells can interplay with other immunocytes, such as T cells, DCs, macrophages, and natural killer (NK) cells and further influence their functions [63]. Another B-cell action is the direct Th cytotoxicity in killing immuno-inhibitory cells and tumour cells. The mechanism of action is the expression, by some B lymphocytes, of the death-inducing molecule Fas ligand (FasL), which kills cells directly [73,74].

Despite these effects of B cells that inhibit tumour development, it was shown that B cells have functions in suppressing immune response through several ways, with Bregs being the dominant element [75]. Indeed, Bregs seem to attenuate immune response in cancers [76,77], but also in other inflammatory diseases [78], activated through the TLR, BCR, CD40/CD40L or B-cell activating factor (BAFF) -signaling pathways. Their immunosuppressive action results also by secreting IL-10, IL-35 and TGF-β cytokines.

In MPM, Anraku et al. [79] studied 32 formalin fixed and paraffine-embedded (FFPE) tissue samples collected from patients who underwent neo-adjuvant chemotherapy followed by extrapleural pneumonectomy. On these samples, the mean counts of positive TILs was: CD4+ = 119.9 ± 94.2, CD8+ = 73.1 ± 40.2, CD25+ = 17.5 ± 12.6, FOXP3+ = 21.8 ± 19 and CD45RO+ = 115.7 ± 56.2 [79]. Pasello et al. [80] observed in chemonaive MPM samples that sarcomatoid/biphasic MPM were characterized by high CD8+ TILs, whereas epithelioid MPM expressed higher peri-tumoral CD4+ T-cells and CD20+ B cells. On the contrary, the analysis of 88 MPM patient samples who underwent chemotherapy or extended pleurectomy/decortication alone or in a context of multimodality treatment, revealed that CD19+ B-cells and CD4+ stromal TILs were significantly enhanced (respectively, *p* = 0.02 and *p* = 0.01) in sarcomatoid MPM [62]. CD8+ stromal TILs were more highly expressed, but without significance in the case of epithelioid MPM [62].

**Figure 5:**
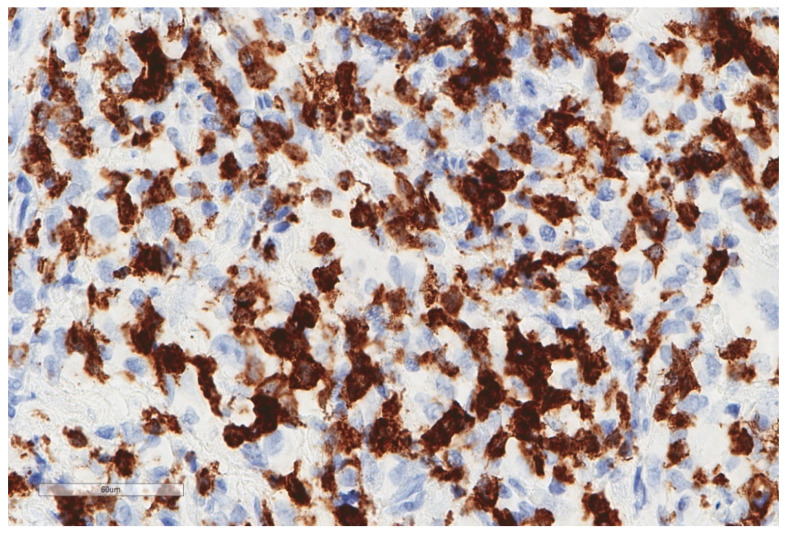
Heavy CD8 intratumoral infiltrate in the malignant mesothelioma case shown in Figure 1 (X400).

Moreover, it has been reported that TILs are associated with prognosis in MPM. Indeed, in 1982, Leigh and Webster were the first to report the importance of lymphocytic infiltration on clinical outcome, although phenotypic analyses of lymphocytes were not performed in their experiments [81]. Among the sub-type populations of TILs, CD8+ TILs (Figure 5) seem to be particularly relevant.

**Figure 6:**
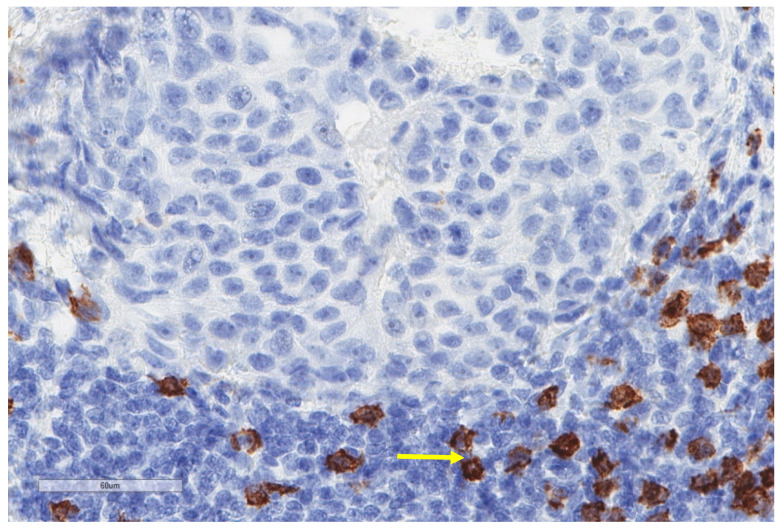
In comparison to the previous case, this malignant mesothelioma harbors few CD8+ lymphocytes (arrow), mostly in the peritumoral compartment (X400).

First, Anraku et al. observed that MPM patients with mediastinal node disease have low levels of CD8+ TILs [79]. Then, apoptotic tumour cells were significantly more present in tumours with high levels of CD8+ TILs in comparison with those with lower level (*p* = 0.02). In particular, high levels of CD8+ TILs were found to significantly increase PFS (adjusted HR (95% CI) 0.38 (0.09–0.87); *p* = 0.02) and OS (adjusted HR (95% CI) 0.39 (0.09–0.89); *p* = 0.02) in tumour samples of patients who underwent surgical resection [79]. Such results were confirmed by Yamada et al. and, thus, highlight that CD8+ TILs (Figure 6) can be used as a prognosis biomarker for patients who underwent extrapleural pneumonectomy for an MPM [82].

Although high CD8+ TILs expression seems an interesting prognostic biomarker in surgically treated patients, its prognostic value remains unclear as contradictory results have been found, especially in chemotherapy-treated patients. Indeed, Losi et al. observed a trend between low lymphocytic infiltration at diagnosis as well as high CD8+ TILs expression and poorer prognosis [83]. Chee et al. [84] performed a tissue micro-array analysis of 302 samples. Their results showed that in the epithelioid group, high CD4+, CD20+ and low FOXP3+ and NP57+ are associated with a better outcome, whereas, in the non-epithelioid group, only low FOXP3 expression was significant [84]. Similarly, in sub-group analysis of patients who were treated with chemotherapy, high CD4+ TILs count was associated with a better prognosis (*p* = 0.034). Therefore, high CD4+ expression seems to be associated with a better response to chemotherapy compared to CD8+ expression. Overall, the authors showed that CD4/CD8 > 1 was associated with a longer survival, only in the epithelioid group (*p* = 0.047) [84]. Similarly, Fusco et al. also observed that stromal CD4/CD8 TILs ratio is a prognostic factor in MPM independently of the histology [62]. They also showed that peritumoral TILs seem to be important to take into account, as high peritumoral CD8+ TILs were associated with a longer survival to chemotherapy (OS > 1 year) and more especially in PD-L1 negative group [62].

In the case of CD20 expression, Ujiie et al. [38] found a similar result as previously described by Chee et al. [84]. Indeed, a high density of CD20 expression (adjusted HR (95% CI) 0.69 (0.51–0.93); *p* = 0.015) was significantly associated with a better prognosis in epithelioid mesothelioma [38]. Such results are consistent with other findings in several cancers highlighting that B-lymphocytes infiltration is associated with a better outcome [35]. However, several studies found low B-lymphocytes expression in MPM [35].

Although helper T-cells and cytotoxic T-cells seem to be highly expressed in the case of MPM, their immune function is decreased by high infiltration of Tregs. Indeed, Hegmans et al. found high level of CD4 + CD25 + FOXP3+ Tregs in the vicinity of the tumour of human MPM samples [33]. Similarly, Marcq et al. found Tregs in 72% of samples of pre-treated or chemotherapy naïve patients [40]. The relevant role of these cells in anti-tumour response was assessed using a mouse model. Indeed, in this in vivo model, the removal of these cells using a CD-25 depleting antibody improved the median survival from 19 to 33 days [33]. In addition, in patients with resectable MPM included in the SMART trial (Surgery for Mesothelioma after Radiation Therapy) [85] evaluating a short, accelerated course of high-dose hemithoracic intensity-modulated radiation therapy followed by extrapleural pneumonectomy, FOXP3+ Tregs were found to be increased after irradiation and, thus, could contribute to limiting its efficiency [86]. More interestingly, in a transgenic DEREG xenograft model (selective depletion of Tregs using diphtheria toxin injection), the transient depletion of FOXP3+ Tregs, following nonablative oligofractionated irradiation, improved the anti-tumour effect. In this model, depletion of FOXP3 was associated with upregulation of immune checkpoint expression (PD-1 and CTLA-4) on CD4+ and CD8+ TILs and a central memory T-cells pattern (CD44 + CD62L + CD4+ T-cells and CD44 + CD62L + CD8+ T-cells) [86].

Similarly, to inflammatory cells, TILs expression can be influenced by chemotherapy in MPM. Pasello et al. performed an analysis of paired samples before and after chemotherapy with cisplatin-pemetrexed. After chemotherapy, there was a significant increase in CD3+ T-cells (*p* = 0.001) and a tendency for increasing of CD68+ macrophages, although not significant. CD8+ TILs were also significantly increased after chemotherapy in comparison with their expression in naive samples [80].

Finally, it was demonstrated that different localisations of MPM harbour various expression of TILs [43]. Kiyotani et al. [87] performed whole-exome sequencing of tumours originated from three different regions (anterior, posterior and diaphragmatic) of MPM patients who underwent surgical resection. They performed TCR repertoire analysis of TILs as well as expression of immune-related genes such as CD4, CD8 and FOXP3 [87]. This whole-exome sequencing revealed different patterns among the three regions of immune-related genes, and the clonality of TILs was also different, as assessed by different diversity indexes of TCR [87]. Such findings highlight intratumoral heterogeneity [87]. In addition, Lievense et al. compared the immune compositions of pleural effusions and tumour biopsies from five patients with an MPM [88]. The authors found that the immune compositions were different, with higher amounts of CD8+ TILs in pleural effusions compared to tumour biopsies, whereas M2-TAMs were more expressed in tumour biopsies compared to pleural effusions [88]. In a larger cohort, Salaroglio et al. observed that the expression of CD8+ cells, Tregs, M2-macrophages as well as immune checkpoint expression in MPM pleural effusion was not associated with patients’ outcome [37]. On the contrary, high intra-tumour Tregs, as well as high expression of the immune checkpoint on pleural tissue, were correlated with a shorter OS [37]. This highlights intra-tumoral heterogeneity and the fact that intratumour immune infiltrate, rather than immune population, is highly predictive of MPM prognosis in pleural effusion.

Overall, these studies show that the MPM microenvironment is highly infiltrated by TILs (Table 2). Anti-tumour activity of CD4+ TILs and CD8+ TILs is decreased by the presence of Tregs. Finally, TILs might be used as prognostic biomarkers, although larger studies are needed.

### 3.2. Exhausted T-Cells

Exhausted T-cells or anergic T-cells are T-cells that are functionally deficient. These cells are described in the concept of immunoediting, which reflects anti-tumour response in three phases: elimination, equilibrium and escape [89]. In the escape phase, tumour cells can escape the immune surveillance through different mechanisms: (1) antigen loss variants or loss of MHC type I expression so that tumour cells are no longer recognized by adaptative immune cells; (2) insensitivity to immune effector mechanisms through the expression of anti-apoptotic molecules; (3) induction of an immunosuppressive microenvironment through the secretion of cytokines and checkpoints on their membrane surface so that tumour cells inhibit T-cells’ activity [89].

As previously mentioned, the MPM microenvironment is characterized by T-cells’ infiltration. In an immunohistochemistry analysis of 67 patients who underwent pleurectomy-decortication for an MPM, Ahmadzada et al. observed that most patients had inflamed immune tumour with CD3 expression (CD3+ positive ≥1%; 75% of patients) within the tumour [90]. Interestingly, the authors reported a significant association between TILs and Bridging integrator 1 (BIN1) (*p* < 0.01) which is a member of Bin–Amphiphysin–Rvs (BAR) domain superfamily involved in cell division and cell migration. BIN1 has also been found to inhibit pro-oncogenic properties of c-Myc. In this analysis, high expression of BIN1 was significantly associated with a better prognosis (adjusted HR (95% CI) 0.39 (0.18–0.82); *p* = 0.01) [90]. As a result, such findings reveal that BIN1 might play an anti-tumour role although its functions might be decreased through interactions with immune microenvironment. Moreover, a correlation was observed between BIN1 expression and TILs, therefore highlighting that T-cell functions could also be suppressed, leading to exhausted T-cells [90]. In this setting, Marcq et al. showed that CD8+ T-cells and helper CD4+ T-cells display markers of exhausted T-cells (i.e., PD-1, LAG-3 and TIM-3) in flow-cytometry analysis of pleural effusion [45]. In pleural effusion, NKs express functional PD-1 [57]. In addition, peripheral blood NKs have been found to express TIM-3 [54].

Such findings highlight that apart from Tregs, which decrease the activity of effector T-cells, the microenvironment also contributes to immune suppression through chronic inflammation and the expression of immune checkpoints.

### 3.3. Neutrophil-to-Lymphocyte Ratio (NLR), Platelet-to-Lymphocyte Ratio (PLR) and Lymphocyte -to-Monocyte Ratio (LMR)

Secondary to asbestos exposure, chronic inflammation of the pleura is involved in MPM carcinogenesis. In addition, systemic inflammation is important to consider as it has been reported to be associated with poor prognosis in several tumours [91] and it is estimated that approximately 30% of patients with MPM experienced symptoms related to systemic inflammation such as fever, weight loss, asthenia and night sweats [92]. Moreover, high NLR has been found to be associated with shorter survival in patients presenting pleural effusion revealing malignancy (*p* = 0.03) [93]. Several composite scores such as those of the European Organisation for Research and Treatment of Cancer (EORTC) and Cancer and Leukemia Group B (CALGB) highlighted that leucocytosis, thrombocytosis and elevated lactate dehydrogenase (LDH) were associated with shorter OS [92]. Therefore, the assessment of the NLR, PLR and lymphocyte-to-monocyte ratio (LMR) have to be taken into account as they can be easily used in clinical practice. Elevated neutrophil and platelet counts are associated with systemic inflammation, whereas decreased lymphocyte count reflects immunosuppression.

High NLR at baseline has been found to be associated with a poor prognosis among 173 patients with an MPM previously treated with systemic chemotherapy (i.e., 54 patients) or chemotherapy-naïve (i.e., 119 patients) [94]. In this retrospective study, NLR ≥ 5 was considered as an elevated ratio. At baseline, 53 patients had an elevated NLR in the chemotherapy-naïve group, whereas 20 patients presented an elevated NLR in the previously treated group [94]. Among all patients, NLR < 5 was significantly associated with a better survival (adjusted HR (95% CI) 2.7 (1.8–3.9); *p* < 0.001). NLR remained a prognostic factor in both chemotherapy-naïve and previously treated patients [94]. Interestingly, NLR was analysed for 65 patients after one cycle of chemotherapy. Among them, 43% of patients presented a normalization of NLR after one cycle of systemic chemotherapy, which was associated with a better prognosis in comparison with patients who presented persistently elevated NLR (median OS 7.8 months and 5.0 months, respectively, *p* = 0.034) [94]. According to these results, NLR ≥ 3 was a significantly independent biomarker associated with a shorter survival among 85 patients who underwent extrapleural pneumonectomy (adjusted HR (95% CI) 1.79 (1.04–3.07); *p* < 0.04) [95]. In 2017, a meta-analysis that involved 11 studies dealing with 1533 patients affected by MPM confirmed these results. Indeed, it was shown that elevated NLR is associated with a poor OS (adjusted HR (95% CI) 1.48 (1.16–1.89); *p* < 0.001). Consistently with findings on OS, it was observed that this elevated ratio was significantly higher in the non-epithelioid group (adjusted OR (95% CI) 0.59 (0.40–0.86); *p = 0*.005) [91].

Tagawa et al. [96], in 65 available blood samples of 85 patients who underwent extrapleural pneumonectomy for MPM, showed in multivariate analysis that PLR < 215 was significantly associated with a longer survival in comparison with patients who presented PLR ≥ 215 at baseline (adjusted HR (95% CI) 0.50 (0.24–0.99); *p* = 0.049). On the contrary, NLR was not identified as an independent prognostic biomarker [96]. Similarly, among 36 blood samples of patients diagnosed with MPM, mean PLR at diagnosis was 225.5 +/− 134.5 and mean NLR was 4.78 +/− 4.50. PLR was also a significant prognostic factor associated with OS [97], in accordance with Tagawa’s et al. results [96]. Such correlation was not found for NLR (area under the ROC curve: 0.599 and 0.749 for NLR and PLR, respectively; *p* = 0.631 and 0.044, respectively) [97].

Yamagishi et al. were interested in blood samples of 150 consecutive patients diagnosed with MPM [98]. Most of the patients (74.7%) in this cohort had NLR ≤ 5, 70.7% of the patients had PLR ≥ 150 and 72.7% had LMR ≥ 2.74. In a multivariate analysis, LMR ≥ 2.74 was significantly associated with a better OS compared to patients with LMR < 2.74 (adjusted HR (95% CI) 2.34 (1.58–3.47); *p* = 0.0001). Thus, the authors showed that LMR is an independent prognostic biomarker in MPM [98].

Taken together, these studies highlight that NLR, PLR and LMR could be useful prognostic biomarkers (Table 3). Evidence suggests that high NLR is associated with a poorer prognosis, although there are contradictory findings [94,95,96,97,98]. PLR might be more interesting, compared to NLR, as it seems that it is less influenced by other factors such as sepsis. Further studies are needed to obtain precise prognostic values of these markers in clinical practice.

## 4. Immune Checkpoints

Immune checkpoint pathways are involved in self-tolerance and prevent tissue from damage causes by excessive immune response [99]. In the setting of immunoediting, tumour cells are able to escape immune response through the expression of checkpoints ligands in the tumour microenvironment [89,99]. Indeed, independently of the inflammatory microenvironment, it has been demonstrated that some tumour cells might be able to express checkpoint ligands on their surface through the activation of a constitutive signalling pathway, such as Akt. On the other hand, checkpoint ligands could be induced as a mechanism of resistance to adaptative immune responses; this occurs in an inflammatory microenvironment [99].

### 4.1. Programmed Cell Death-1 (PD-1) and PD Ligand-1 (PD-L1)

Immune checkpoint inhibitors that target programmed cell death protein 1 (PD-1) or PD1 ligand 1 (PD-L1) have demonstrated activity in relapsed mesothelioma and are undergoing further evaluation.

A case series of 68 FFPE pleural samples reported PD-1 expression on 62% of TILs, although the percentage of positive cells was low (i.e., mean percentage of positive cells of 9%). The expression of PD-1 in TILs was significantly associated with the expression of CD3 and CD8 (*p* = 0.001 and 0.037, respectively) [100]. Similarly, PD-1 was expressed on 65% TILs of unpretreated MPM and on 71% of pretreated MPM with chemotherapy [40]. The expression of PD-1 on TILs was significantly correlated with cells expressing CD4 + FOXP3+ and granzyme B [40]. In the case of tumour cells, PD-1 expression was found in only 10% of unpretreated samples [40]. Analysis of 54 malignant pleural effusions (33 from MPM) revealed that both NK and Th17 cells express functional PD-1, thus leading to a decrease in their function [57].

**Figure 7:**
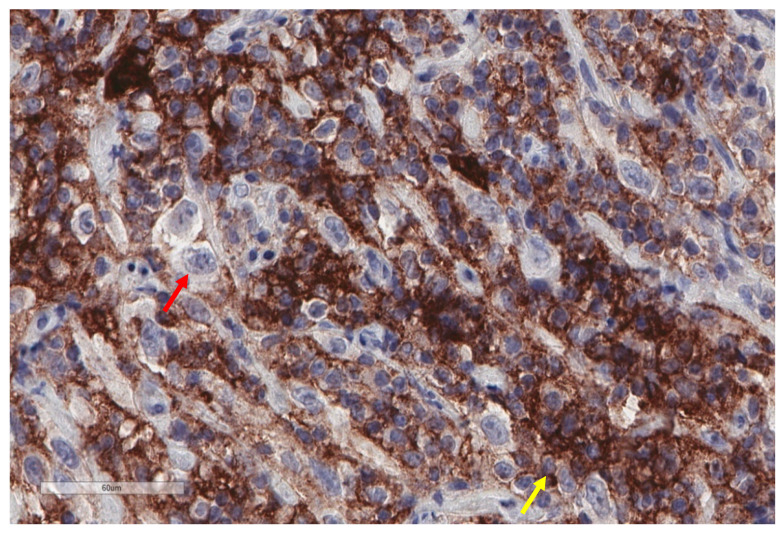
Abundant PD-L1 expression in this case of malignant mesothelioma (X400). Most stained cells probably correspond to immune cells (smaller size, less atypia; yellow arrow), while most tumour cells show no membranous staining (red arrow). However, further markers in conjunction to PD-L1 would be necessary to better characterize the two cell populations.

PD-L1 interacts with PD-1 receptor on T cells and inhibits their function, leading to anergic T cells. PD-L1 has been found to be highly expressed in MPM cells and associated with a poorer prognosis [101]. Indeed, immunohistochemistry analysis (Figure 7) of 106 patient samples with an MPM, showed that 40% of them expressed PD-L1 ≥ 5% [102]. In particular, all sarcomatoid MPM (*n* = 17) expressed PD-L1, except in one case. On the contrary, in the PD-L1 positive group, the epithelioid sub-type was significantly less represented in comparison with the PD-L1 negative group (33% and 84%, respectively; *p* < 0.0001) [102].

Among all patients, OS was significantly shorter in PD-L1 positive group in comparison with PD-L1 negative group (OS of 5 months and 14.5 months, respectively; *p* < 0.0001). According to histologic sub-group analysis, PD-L1 expression was significantly associated with a worse prognosis in sarcomatoid mesotheliomas (adjusted risk ratio (RR) (95% CI) 2.18 (1.08–4.23); *p* = 0.03) [102]. Consistently with Mansfield et al., another study highlighted that PD-L1 expression is associated with a poorer prognosis, especially in the case of non-epithelioid mesotheliomas [103]. Similar results were also found according to Cedres et al., although PD-L1 expression (i.e., ≥1%) was observed to a lesser extent (20.7% among 77 patient samples analysis) [103]. More recently, Rrapaj et al. found that PD-L1 was expressed (i.e., ≥5%) in 12.12% of 198 MPM patient samples [104]. In line with previous results, PD-L1 expression was associated with poorer prognosis, particularly in the case of the Eastern Cooperative Oncology Group (ECOG), with a performance status ≥2 (adjusted HR (95% CI) 2.3077 (1.264–4.212); *p* = 0.0064) [104].

Contradictory data were found by Awad et al. [105]; in their study, there was no significant difference in OS according to PD-L1 expression status. The authors analysed 43 resected MPM samples (with or without neoadjuvant chemotherapy before surgical resection) that have been dissociated into single-cell suspensions in order to assess immune cell phenotyping according to PD-L1 expression status (i.e., PD-L1 ≥ 1%). CD45, a pan-leucocyte marker, was significantly more expressed in PD-L1 positive tumours compared to PD-L1 negative tumours (median 87.7% and 68.2%, respectively; *p* = 0.05). Consistently with this result, CD45 was also significantly more highly expressed in the non-epithelioid group. Among CD45+ immune cells, CD3+ T-cells were significantly more expressed in PD-L1 positive group tumours and non-epithelioid mesotheliomas. No significant difference according to PD-L1 expression status was found in other sub-populations of immune cells (CD19 B cells, CD66b neutrophils, CD123 DCs, CD56 NKs and CD33 monocytes) as well as in the proportion of CD4 and CD8 cells. Interestingly, CDR45RA + CCR7^−^ effector CD4+ cells, which represent T central memory cells, were significantly less expressed in PD-L1 positive tumours compared to PD-L1 negative tumours (*p* = 0.01). In the PD-L1 positive group, CD4 T cells expressed higher amounts of FOXP3 marker (*p* = 0.005). Similar to CD4 T cells, a significantly higher proportion of CD8 memory T cells was found in the PD-L1 positive group [105]. Similarly, Losi et al. observed, in an analysis of 55 FFPE MPM samples, that high PD-L1 expression (≥50%) tends towards a poorer prognosis, although not significantly [83]. Likewise, higher immune score (2+/3+) was significantly (*p* = 0.019) associated with intermediate PD-L1 expression (1–49%) and a high infiltrate of CD4+ TILs was observed independently of PD-L1 status [83]. Conversely with Awad et al. [105] and Losi et al. [83], recent evidence suggests that CD4+ (*p* = 0.01) and CD19+ (*p* = 0.04) stromal TILs are significantly more highly expressed in the case of PD-L1 positive MPM compared to PD-L1 negative MPM [62]. Moreover, in PD-L1 negative MPM, CD4/CD8 > 1 stromal TILs, alone or in association with high CD19 stromal TILs, were associated with a better outcome (*p* = 0.03) [62]. On the contrary, it seems that in PD-L1 positive tumours, high stromal CD8+ TILs expression is associated with a poorer survival (*p* = 0.007) [62].

PD-L1 was found to be less expressed in pleural effusion in comparison with pleural tissues (36% and 46%, respectively, for a PD-L1 ≥ 1%) [106]. Despite lesser expression in cytologic samples, the authors found a moderate concordance in PD-L1 ≥ 1% expression between histologic and cytologic results in the case of epithelioid MPM (Cohen’s κ coefficient (95% CI) 0.43 (0.16–0.69)). On the contrary, there was no concordance in the case of non-epithelioid MPM. In addition, the concordance in PD-L1 expression between pleural tissues and pleural effusions decreased with the level of cut-off for PD-L1 expression, which highlights intra-tumoral heterogeneity [106].

### 4.2. Expression of Other Immune Checkpoints

T cell immunoglobulin mucin-3 (TIM-3) and lymphocyte activation gene-3 (LAG-3) are other immune checkpoints. TIM-3 is expressed on the surface of immune cells and interaction with its ligand—galectin-9—decreased immune cells’ functioning [107]. LAG-3 is expressed on the surface of activated T cells and is implicated in T cell exhaustion [108]. TIM-3 has been found to be expressed on tumour cells and TILs of unpretreated and chemotherapy pretreated MPM samples [40]. As an assessment of immunotolerance in the microenvironment, a strong correlation was found between the expression of TIM-3 and PDL-1 on TILs in the stroma (RR = 0.48; *p* < 0.001) [40]. Moreover, TIM-3+ lymphocytes in lymphoid aggregates (a lymphoid aggregate is defined as 50 or more lymphocytes clustered together) were strongly correlated with TIM-3 TILs (RR = 0.64; *p* < 0.001) and CD4 TILs (RR = 0.42; *p* = 0.010) in the stroma [40]. Interestingly, TIM-3 could be used as a prognostic biomarker, independently of chemotherapy, as OS was better for patients with high TIM-3 expression in aggregate samples (RR = 0.47; *p* = 0.002) [40]. Such findings of TIM-3 involvement in the immune microenvironment of MPM were also found on pleural and ascites fluid samples from MPM patients who were treated with chemotherapy [45]. Of note, TIM-3 was found to be particularly expressed on NK cells and less expressed on CD4+ and CD8+ T-cells, leading to exhausted cells [45].

Contrary to TIM-3, no expression of LAG-3 was found in unpretreated and chemotherapy pretreated MPM samples [40]. Marcq et al. found LAG-3 expression in pleural and ascites fluids of patients’ samples pretreated with chemotherapy; LAG-3 was preferentially expressed in NK cells [45]. Conversely, Salaroglio et al. found expression of LAG-3 in both pleural effusion and pleural tissue of MPM patients [37].

B7 homolog 3 (B7-H3), also known as CD276, belongs to the B7 family of immune checkpoint proteins [109,110]. B7-H3 is a transmembrane protein that has been found to be expressed in tumour cells, antigen presenting cells and NKs. Although B7-H3 receptors have not been identified, it is thought that B7-H3 negatively regulates T helper (Th-1) and Th-2 mediated responses. In this context, several studies outlined that B7-H3 is upregulated in various types of cancers and associated with a poorer prognosis [109,110]. Using immunohistochemistry analysis from 31 patients with an MPM, including eight patients previously treated with chemotherapy, Matsumura et al. showed that B7-H3 was highly expressed (B7-H3 ≥ 1%) in both epithelioid and non-epithelioid sub-types (90.9% and 88.9%, respectively) [111]. Moreover, it was found that B7-H3 was significantly more expressed in comparison with PD-L1 in the epithelioid sub-type (*p* < 0.00001). Such a correlation was not found in non-epithelioid MPM. In particular, the authors showed that among 13 positive PD-L1 samples, 12 of them were also positive for B7-H3 [111]. Such findings underlie promising interest for combined immunotherapy.

Cytotoxic T-lymphocyte antigen 4 (CTLA-4) is another immune checkpoint expressed on effector T-cells. It interacts with CD80/CD86 on antigen-presenting cells and, thus, blocks the binding to CD28 costimulatory molecule [112,113]. CTLA-4 is also expressed on the surface of T-regs [114]. Besides, a soluble isoform of CTLA-4 has been described and can be secreted by both effector T-cells and T-regs [115]. There are few reports about CTLA-4 expression in MPM. Immunohistochemistry analysis at a baseline of 41 MPM samples revealed that CTLA-4 was expressed in 56% of cases, with a proportion of positive cells that was highly variable (ranging from 10 to 95%) [116]. In addition, there was a trend towards higher expression of CTLA-4 in epithelioid sub-type. Soluble isoform of CTLA-4 was also studied in the blood samples and pleural effusions on these patients [116]. Both serum and pleural effusion were found to express soluble CTLA-4 (Pearson’s correlation coefficient of 0.52). This isoform was more expressed in blood samples in comparison with pleural effusion (*p* = 0.020), in different histology sub-types, with statistical significance for sarcomatoid MPM (*p* = 0.010) [116]. High CTLA-4 expression in pleural effusions, biopsy and blood samples was found to be associated with a better OS compared to patients with low expression of CTLA-4. High expression of soluble isoform of CTLA-4 in pleural effusions (>67 pg/mL) was significantly associated with a prolonged survival (adjusted HR (95% CI) 0.36 (0.17–0.76); *p* = 0.007) [116].

Recently, a new area of investigation in immune checkpoints emerged in MPM. V-domain Ig suppressor of T-cell activation (VISTA) is a novel immune checkpoint gene that is structurally similar to PD-L1. VISTA is expressed on hematopoietic cells and T-lymphocytes and, when overexpressed, suppresses early T-cell activation and proliferation and reduces cytokine production [117,118]. VISTA acts as both a ligand on antigen-presenting cells and as a receptor on T-cells [119,120]. Increased VISTA expression has been observed in tumour cells and/or immune microenvironments of some malignant tumours [119,120]. VSIG- 3 was recently discovered as the ligand for VISTA [121].

In a recent comprehensive integrated genomic study in MPM, The Cancer Genome Atlas Research Network found that epithelioid tumours display the highest expression of VISTA among all investigated cancers [122], suggesting that VISTA might be a potential therapeutic target in MPM.

**Figure 8:**
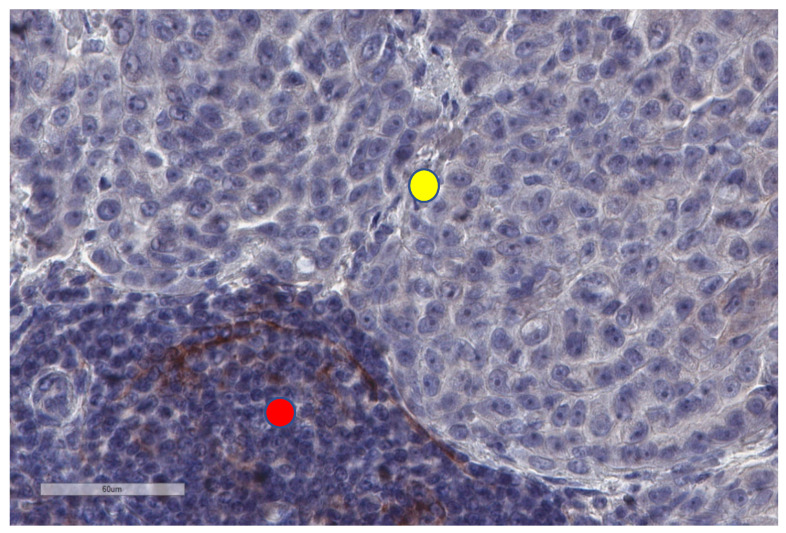
PD-L1 is not expressed by tumour cells (upper part of the image, yellow circle) in this malignant mesothelioma, but there are a few PD-L1+ immune cells in the neighbouring stroma (lower part, red circle).

In a large MPM cohort (254 epithelioid, 24 biphasic and 41 sarcomatoid), VISTA expression was found in 88% of epithelioid, 90% of biphasic and 42% of sarcomatoid tumours [123]. Overall, the VISTA score was significantly higher in epithelioid compared to non-epthelioid tumours (*p* < 0.001). On the contrary, the same study reported expression of PD-L1, respectively, in 33% of epithelioid, 43% of biphasic and 75% of sarcomatoid tumours, and the PD-L1 score was significantly higher for sarcomatoid compared to non sarcomatoid tumours (*p* < 0.001). VISTA and PD-L1 were expressed in inflammatory cells in 94% (*n* = 317) and 24% (*n* = 303) of mesothelioma (Figure 8), respectively. Optimal prognostic cutoffs for VISTA and PD-L1 were 40% and 30%, respectively. In multivariable analysis, VISTA and PD-L1 expression in mesothelioma were associated with better and worse overall survival (*p* = 0.001 and *p* = 0.002), respectively, independent of histology. The authors concluded that these findings may explain poor responses to anti-PD-(L)1 immunotherapy and suggest VISTA as a potential novel target in pleural mesothelioma [123].

These studies highlight those immune checkpoints are expressed in MPM (Table 4) and, consequently, constitute interesting targets to restore immune response against the tumour.

## 5. Novel Insight in the Treatment of Malignant Pleural Mesothelioma

### 5.1. Immune Checkpoints Inhibitors (ICIs)

Immunotherapy based on ICIs is a promising approach to improve outcome of patients with an MPM, although only few patients seem to benefit from this innovative treatment. As a consequence, transcriptomic analysis could be used to define the profile of patients who could better respond to immunotherapy. Alcala et al. performed a transcriptomic analysis of 284 MPM samples and identified three main transcriptomic profiles [124]. Firstly, “hot” tumours, characterized by high infiltration of T-lymphocytes, high expression of immune checkpoints (PD(L)1, CTLA-4, TIM-3 and LAG-3) and proangiogenic genes (VEGFR1, VEGFR3 and PDGFRB), were identified. These “hot” tumours are mainly encountered in non-epithelioid MPM and are associated with a short median survival (7 months) [124]. The second profile is defined by high expression levels of VEGFR2 and VISTA, enriched for epithelioid MPM. This immune profile is associated with a better prognosis, with a median OS of 36 months. Finally, the third transcriptomic profile is represented by “cold” tumours with a lack of immune effector cells and high expression of proangiogenic genes (VEGFR1, VEGFR3 and PDGFRB). This profile is represented in non-epithelioid MPM and associated with a poor survival (median OS of 10 months) [124]. Moreover, proof-of-concept of the promising possibility of using ICIs in mesothelioma was established a few years ago; for instance, the ability of avelumab to mediate antibody-dependent cellular cytotoxicity in in vitro experiments, as well as the blocking of the PD-1/PD-L1 pathway to restore immune response [125].

In this setting, several clinical trials studying immune checkpoint inhibitors (ICIs), alone or in association with chemotherapy, have already been conducted. Promising results have been observed with immunotherapy, although no ICIs have yet been approved. In particular, a randomised phase II MAPS II trial, which enrolled patients in nivolumab + ipilimumab (anti-PD-1 antibody and anti-CTLA-4 antibody, respectively) in combination vs. nivolumab alone, as a second or third-line treatment, showed interesting results with a median OS of 15.9 vs. 11.9 months, respectively [126]. More recently, the results of the phase III CHECKMATE 743 trial were reported, showing a significant improvement of OS in the nivolumab + ipilimumab group compared to the standard chemotherapy group as first-line treatment (median OS 18.1 vs. 14.1 months, respectively, HR = 0.74, *p* = 0.002) [127]. In addition, the association of chemotherapy with ICIs could be particularly relevant in the case of MPM. Indeed, CD8+ TILs have been reported to increase after chemotherapy [80]. Therefore, increasing the neo-antigen load with chemotherapy seems promising in order to enhance the anti-tumour response with the use of ICIs. Moreover, in vitro experiments showed that chemotherapy could influence the expression of immune checkpoints. Indeed, Marcq et al. co-cultured MPM cell lines (NCI-H2818 and NCI-H2795 epithelioid cell lines, NCI-H2731 sarcomatoid cell line) with healthy donor peripheral blood mononuclear cells to study the impact of chemotherapy on immune checkpoint expression [128]. The results of this study showed a decreasing trend of immune checkpoint expression (PD-1, PD-L1, PD-L2, TIM-3, LAG-3 and galectin-9) in two of the three cell lines when treated with cisplatin, oxaliplatin or pemetrexed [128].

As observed in other cancers, it seems that a sub-group of patients may significantly benefit from treatment with ICIs, so the aim is to develop predictive biomarkers of response to immunotherapy. In the case of MPM, it seems that PD-L1 is not a good predictive biomarker, as shown by the reported results of MAPS II and Checkmate 743 [126,127]. As shown above, transcriptomic analysis is interesting to define the profiles of tumours that could respond better to immunotherapy [124], although it seems difficult to perform in routine clinical practice. Finally, as MPM is characterized by a low average tumour mutational burden (TMB), TMB cannot be used as a predictive biomarker of response to immunotherapy [129,130]. More recently, evidence suggests that BAP-1 loss is associated with signatures of cytokine signalling and of the innate immune system in genomic and transcriptomic analysis of 19 peritoneal mesothelioma [131]. Similar results have been observed in the case of MPM, as BAP-1 loss tumours were associated with a trend towards higher expression of PD-L1 and a significant association with the mRNA signature of activated DCs [132]. Thus, BAP-1 loss tumours are associated with an inflammatory tumour microenvironment and may respond better to immunotherapy. Finally, another interesting biomarker of response to immunotherapy is the analysis of the microbiome [129].

#### Dendritic Cell Immunotherapy

As previously explained, patients with MPM have low amounts of DCs. Moreover, DCs are functionally deficient. Pulsed DCs have shown interesting results in pre-clinical models as they prevented tumour outgrowth [44]. Therefore, dendritic cell immunotherapy is a promising approach as it stimulates effector T-cells, which might be exhausted in MPM. In this setting, Cornelissen et al. reported interesting results in 10 patients with MPM [133]. In this study, they used autologous monocyte-derived DCs loaded with autologous tumour cell lysate [133]. The authors associated this treatment with metronomic cyclophosphamide [133], as it was previously reported that metronomic cyclophosphamide reduced the number of Tregs in murine models [134]. Therefore, five patients received dendritic cell immunotherapy after platin-based chemotherapy, while five other patients received the treatment after a multimodal strategy of induction chemotherapy and pleurectomy-decortication [133]. Interestingly, it was shown that disease control was achieved in 8 out of 10 patients, while 7 patients had OS ≥ 24 months. Moreover, this therapy was safe as no grade 3/4 toxicity was reported. As in preclinical models, the number of Tregs decreased in peripheral blood after the first week of metronomic cyclophosphamide (*p* = 0.02) [133]. More recently, promising results were also reported using autologous monocyte-derived DCs pulsed with allogeneic tumour lysates from mesothelioma cell lines [135]. In this first-in-human trial, median OS was not reached (median follow-up of 22.8 months) among nine patients (five patients were treated as maintenance after chemotherapy and four patients were treatment-naïve) [135]. Actually, a phase II/III trial (DENdritic cell Immunotherapy for Mesothelioma—DENIM trial) is currently ongoing to evaluate DCs immunotherapy vs. the best supportive care in maintenance therapy after standard chemotherapy [136].

### 5.2. Adoptive T-Cell Therapy

This innovative therapy uses engineered T cells prepared ex vivo directed towards tumour antigens, which leads to chimeric antigen receptor (CAR) T cells. In the case of MPM, these novel therapeutic strategies target mesothelin [137,138]. Mesothelin is a tumour-associated antigen that is significantly expressed in tumour biopsies of patients with MPM, whereas it has no or low levels of expression in healthy mesothelioma cells [137,138]. Actually, the on-going trials are phase I/II clinical trials with intravenous and intrapleural mesothelin CAR T cells. The results showed that this therapy is safe although response rates seem to be relatively low. Future studies will also define the role of CAR T-cells in MPM by improving their design (the ability of CAR T-cells to infiltrate the host tumour, resistance to tumour exhaustion and persistence to prevent tumour recurrence) [137,138].

### 5.3. Exosome-Based Therapy

Exosome-based therapy is actually an approach in the development of targeted therapy to improve drug delivery [139,140]. Exosomes are extra-cellular vesicles (EVs) with a diameter between 20 and 150 nm. EVs are lipid membrane vesicles that are actively secreted by various cell types, including cancer cells, and are characterized by a various cargo composed of nucleic acids, proteins and lipids [139,140]. EVs sources are easy to access through bodily fluids and can be engineered in various ways: (1) pre-existing endogenous cargo is used as the therapeutic molecule; (2) EVs can be engineered with a plasmid to induce the expression of exogenous proteins; (3) direct introduction of a drug into EVs after isolation [139,140].

Several sources of evidence suggest that exosomes are secreted by MPM cells and promote tumour growth and invasion [141,142,143]. In particular, pleural effusions of MPM have been found to be enriched in exosomes [141,142,143]. Greening et al. performed a proteomic analysis on malignant mesothelioma-derived exosomes. This enables the constitution of a molecular signature of malignant-exosome including annexin, heat shock protein, pyruvate kinase, alpha-enolase, glucose6-phosphate1-dehydrogenase and tubulin isotypes [142]. In the context of immune microenvironment, it is interesting to note that exosome might contribute to immune suppression in MPM. Indeed, the incubation of mesothelioma-derived exosome with fresh peripheral blood leucocytes revealed a decreased expression in NKG2D receptor on CD8+ T-cells [144]. MPM-derived exosome in pleural effusion express CD39 and CD73—receptors that are known to produce extracellular adenosine and promote downregulation of T-cells functions—which highlights, as well, that exosome contributes to harness T-cells functions [145]. In addition, PD-L1 expression has been reported in glioblastoma and metastatic melanoma-derived exosomes [141]. It seems that PD-L1 exosomes suppress CD8+ T-cells functions [146]. Thus, to assess PD-L1 in MPM-derived exosomes seem of particular interest as PD-L1 is known to be highly expressed in MPM.

Finally, in vitro studies have shown promising results using exosome-based therapy. Indeed, Munson et al. showed that miR-16-5p, a tumour suppressor, is preferentially secreted by MPM cells through exosomes. The inhibition of exosome secretion, using small molecule inhibitors GW4869 or a combination of Bisindolylmaleimide-I with chloramide with or without cisplatin, significantly restored the expression of miR-16-5p in vitro and leads to MPM cells death [147]. In addition, Mahaweni et al. observed an increased survival in mouse models when using DCs loaded with MPM exosome in comparison with untreated mice or mice treated with tumour lysate-loaded DCs [148].

Overall, in vitro and in vivo experiments in mouse models highlight that exosome are potential molecules to target in order to develop new treatment strategies in MPM.

### 5.4. Stimulator of INterferon Genes (STING) Agonists

STING is a transmembrane protein that is localized in the endoplasmic reticulum [149]. Upon DNA sensing, cyclic GMP-AMP synthase triggers the formation of cyclic GMP-AMP (cGAMP), which, in turn, activated STING. Activated STING interacts with TANK-binding kinase 1 (TBK1). IRF3 transcription factor binds to this complex, which, in turn, induces the activation of targeted genes including interferon-I and inflammatory cytokines [149]. Several studies suggest that STING innate immune pathway can enhance anti-tumour immune response. Indeed, in vivo experiments have shown that STING −/− mice present fast tumour growth and deficiency in IFN-β gene expression by TILs [149,150]. In line with these results, injection of STING agonists using synthetic cyclic dinucleotide derivatives was associated with tumour control growth in xenograft mouse models (sub-cutaneous injection of B16.F10 melanoma cell lines, 4T1 breast cancer cell lines and CT26 colon cancer cell lines) [149,151]. STING pathway activation increased the expression of IFN-β gene expression by TILs, CD86 co-stimulatory molecules on antigen-presenting cells and CD8+ T-cells cross-priming [149,151]. Several clinical trials are currently ongoing in this field, with the development of STING agonists in monotherapy or in combination with other therapies such as ICIs [149,152].

However, STING may also facilitate tumour progression. In comparison with normal tissues, STING expression was significantly upregulated in colorectal carcinoma, renal clear cell carcinoma, stomach adenocarcinoma, and thyroid carcinoma, but was downregulated in lung non-small-cell carcinoma, prostate carcinoma, and endometrial carcinoma [153]. These results suggest that it is necessary to deeply and fully evaluate the functions of STING signalling in cancer immunity and cancer progression before the application of STING agonist-based anticancer immune therapy in routine practice [153,154].

## 6. Conclusions

The immune microenvironment of MPM is complex and could be considered as mainly immunotolerant or immunosuppressive. Moreover, the expression profile of immune cells and immune checkpoints can be different according to histology, and even within the same tumour, reflecting intra-tumoral heterogeneity. It seems that macrophages are involved at an early stage of the immune response following asbestos exposure. Other innate cells such as dendritic or NK cells may also be involved in the immunological microenvironment of MPM, contributing to the escape of the tumour from immune surveillance. The MPM microenvironment is also highly infiltrated by CD4+ and CD8+ TILs. Although high CD4+ TILs seem to be associated with higher response rates in chemotherapy-treated patients, and with better prognosis, the role of CD8+ TILs still remains unclear. Other adaptive cells, such as neutrophils and platelets, that are present in the MPM microenvironment may be interesting biomarkers, especially platelets; however, further studies are needed to assess their prognostic value in routine practice. On the contrary, high intra-tumour Tregs are associated with shorter patients’ survival. In the same way, immune checkpoints are highly expressed in the MPM microenvironment with an adverse effect on the patients’ survival, and consequently, they constitute interesting targets to restore immune response against the tumour. Overall, future research may focus on targeting molecular vulnerabilities associated with the immunotolerant microenvironment of MPM to improve patients’ outcomes.

## Figures and Tables

**Table 1:** Studies assessing the innate immune cells microenvironment of MPM.

Study	n	Study Aim	Method	Results
Burt., 2011 [34]	52	Circulating monocytes, tumour-infiltrating macrophages and OS	Blood and surgical specimen for tissue microarray	Higher number of monocytes was correlated with poor survival in all patients, and high density of macrophages correlated with poor survival in non-epithelial tumours
Salaroglio., 2019 [37]	275	Intrapleural and Intratumor T-regs, M2, MDSC, TILs and OS, PFS	Pleural fluid and pleural biopsies by flow cytometry	MDSC related to anergy of TILs. High intratumor T-regs, MDSC correlated with poor OS/PFS, not observed in pleural fluid
Marcq., 2017 [40]	54	Macrophages (CD68), PD-L1, TIM-3, CD4, CD8, CD45RO	Immunohistochemistry Tissue biopsies	Expression of CD68+ macrophages in the stroma correlates with the number of stromal Tregs
Cornelissen., 2014 [39]	16	Intratumor CD8, total macrophages, M2 and OS	Immunohistochemistry Comparison CT induction + surgery vs. CT alone	CD8+, total and M2 macrophages: no relation to OS; Rate M2/TAMs correlates with poor OS (*p* < 0.05)
Cornwall., 2016 [42]	48	Dendritic cells (DC) MPM vs. controls	Whole blood analysis by flow cytometry	Decreased numbers of DC Reduced ability to process antigen and reduced expression of costimulatory molecules inducing anergised/tolerized T cells.
Sottile., 2019 [54]	27	Circulating NK and T cells before and after treatment with tremelimumab (CTL4)	RNA expression from MPM biopsies	MM patients have perturbed NK cells before CTL4, while, after treatment, NK shift to normal levels
Tumino., 2019 [57]	33	NK cells Innate lymphoid cells (ILC) 1,2,3	Pleural fluid Flow cytometry	NK highly expressed ILC3 more highly expressed than ILC2 and ILC1

**Table 2:** Studies assessing the adaptative immune cells microenvironment of MPM.

Study	n	Study Aim	Method	Results
*Marcq.,* 2017 *[40]*	54	CD68, PD-L1, TIM-3, CD4, CD8, CD45RO	Immunohistochemistry tissue biopsies	High CD4+ is associated with better survival Increase in the CD45RO expression on stromal lymphocytes was significantly associated with a lower likelihood of partial or complete response to chemotherapy
*Fusco.,* 2020 *[62]*	*88*	CD4, CD8, PD-L1 and survival in patients treated with chemotherapy	Tissue biopsies for microarray	Stromal low CD4+ and high CD8+ were correlated with poor survival In PD-L1 CPS > 1, stromal high CD8+ was a poor prognostic factor CD4/CD8 > 1 associated with a longer survival independently of the histologic sub-type
*Pasello*, 2018 *[80]*	*130*	CD4, CD8, CD68, Ki-67, PD-L1 and survival/before and after chemotherapy	Tissue biopsies Immunohistochemistry	CD3+ T-cells and CD8+ TILs significantly increased after chemotherapy High CD8+ were correlated with high macrophages, PD-L1 expression and aggressiveness High CD8 were correlated with poor survival and low response to chemotherapy
*Losi.,* 2019 *[83]*	*55*	Tumor CD4, CD8, PD-L1, and survival	Immunohistochemisty Tissue biopsies	Low lymphocytic TILs expression and high CD8+ TILs at baseline associated with shorter survival
*Anraku.,* 2008 *[79]*	*32*	CD4, CD8, CD25, FOXP3, CD45RO and OS, PFS	Immunohistochemistry in MPM from extrapleural pneumonectomy	High level of CD8+ TILs expression is associated with a better OS/PFS for patients with extrapleural pneumonectomy
*Yamada.,* 2010 *[82]*	*27*	CD4, CD8, NK cells, HLA-1 and OS	Immunohistochemistry in MPM from extrapleural pneumonectomy	High density of CD8+ TILs is independently associated with a significantly better OS in this patient population
*Chee.,* 2017 *[84]*	*302*	CD4, CD8, CD25, CD2O, FOXP3, CD45RO, neutrophils (NP57+), natural killer cells (CD56+) macrophages (CD68+) and OS, PFS	Tissue microarray	High CD4+, CD20+ and low FOXP3 expression were associated with better OS in epithelial tumours Low CD8+ and low FOXP3 expression were associated with better OS in non-epithelial tumours CD4/CD8 > 1 associated with a longer survival in epithelioid tumours
*Ujiie.,* 2015 *[38]*	*230*	TAMs, TILs and survival	Immunohistochemistry. Tissue microarray	High CD163+ TAMs and low CD8+ TILs associated with worse prognosis

**Table 3:** Studies with neutrophil-to-lymphocyte ratio (NLR), platelet-to-lymphocyte ratio (PLR) and lymphocyte-to-monocyte ratio (LMR) as potential prognostic biomarkers in malignant pleural mesothelioma (MPM).

Study	*n*	Study Aim	Method	Results
Chen, 2017 [91]	1533	NLR and overall survival (OS)	Meta-analysis of 11 studies	NLR significantly higher in non-epithelioid group Elevated NLR was associated with a poor OS
Kao, 2010 [94]	173	NLR and OS	Chemotherapy and chemotherapy naïve patients	NLR < 5 associated with better OS in chemotherapy group and chemotherapy-naïve group Normalization of NLR ratio after one cycle of chemotherapy associated with a better OS
Kao, 2011 [95]	85	NLR and OS	Patients with extrapleural pneumonectomy	NLR ≥ 3 was associated with poor OS in patients with extrapleural pneumonectomy
Tagawa, 2015 [96]	65	NLR, PLR and OS	Patients with extrapleural pneumonectomy	NLR ≥ 3.5 was associated with poor OS in univariate analysis PLR ≤ 215 was associated with better OS in both univariate and multivariate analysis
Yamagishi, 2015 [98]	150	NLR, PLR, LMR and OS	Blood samples from patients with MPM at diagnosis	At univariate analysis, NLR > 5, PLR > 150 and LMR < 2.74 were associated with poor OS. Only LMR was independent predictor of survival in multivariate analysis.

**Table 4:** Studies with immune checkpoints expression in MPM.

Study	*n*	Study Aim	Method	Results
Mansfield, 2014 [102]	106	PD-L1 and overall survival (OS)	Tissue biopsy immunohistochemistry PD-L1 > 5%	PD-L1 highly expressed in MPM cells associated with poor OS Patients with PD-L1 highly expressed were less likely to be offered surgery PD-L1 expression associated with poor OS in sarcomatoid MPM
Cedrés, 2015 [103]	77	PD-L1 and overall survival (OS)	Tissue biopsy immunohistochemistry PD-L1 > 1%	PD-L1 positive cases were associated with poor OS
Rrapaj, 2021 [104]	198	PD-L1 and overall survival (OS)	Tissue biopsy Immunohistochemistry PD-L1 > 5%	PD-L1 positive cases were associated with poor OS
Awad, 2016 [105]	43	PD-L1, CD4+, CD8+, TIMP3, CD45+ and overall survival (OS)	Surgically resected MPM Next generation sequencing Flow cytometry	PD-L1 positive cases were associated with CD4+, CD8+, TIM3, CD45+ positive cases No difference in survival according to PD-L1 status
Mansour, 2021 [106]	61	PD-L1 from biopsies vs. pleural effusions	Immunohistochemistry PD-L1 ≥ 1% vs. 5% vs. 10% vs. 50%	PD-L1 is less expressed in pleural effusions compared to pleural tissues Higher concordance for PD-L1 at ≥ 1% cut-off in epithelioid MPM for histologic and cytologic samples
Marcq, 2017 [40]	54	CD68, PD-L1, TIM-3, CD4, CD8, CD45RO	Immunohistochemistry Tissue biopsies	TIM-3 expression is an independent prognostic factor of better survival
Marcq, 2017 [45]	6	PD-1, PD-L1, TIM-3, LAG-3, CD4, CD8, NK	Pleural effusions Flow cytometry	LAG-3 and TIM-3 expressed in pleural effusion on CD4+, CD8+ and NKs
Salaroglio, 2019 [37]	275	Intrapleural and Intratumor T-regs, M2, MDSC, TILs, TIM-3, LAG-3 and OS, PFS	Pleural fluid and pleural biopsies by flow cytometry	LAG-3 expressed in both pleural effusions and pleural tissue Low PD-1+/LAG-3+/TIM-3+ CD4+ TILS were related to better survival in pleural tissue
Matsumura, 2020 [111]	31	PD-L1, B7 homolog 3 (B7-H3)	Immunohistochemistry Tissue biopsies Confirmation of B7-H3 by flow cytometry	B7-H3 highly expressed in chemotherapy-pretreated patients, in both epithelioid and non-epithelioid sub-types B7-H3 significantly more expressed compared to PD-L1 in epithelioid MPM No significant difference in the expression levels of PD-L1 and B7-H3
Roncella, 2016 [116]	45	CTLA-4	Tissue biopsies, blood samples and pleural effusions from patients with MPM	Variable expression (56% cases) in biopsies Higher levels in blood samples compared to pleural effusions Higher levels in tissue for epithelioid tumours Higher levels in blood samples for sarcomatoid tumours High CTLA-4 expression associated with a better survival
